# Will bevacizumab biosimilars impact the value of systemic therapy in gynecologic cancers?

**DOI:** 10.1186/s40661-017-0045-x

**Published:** 2017-03-21

**Authors:** Bradley J. Monk, Warner K. Huh, Julie Ann Rosenberg, Ira Jacobs

**Affiliations:** 1 Arizona Oncology (US Oncology Network), University of Arizona College of Medicine, Creighton University School of Medicine at St. Joseph’s Hospital, Phoenix, AZ USA; 2 University of Alabama at Birmingham, Birmingham, AL USA; 3 Pfizer, Groton, CT USA; 4 Early Oncology Development and Clinical Research, Pfizer, 219 East 42nd Street, New York, NY 10017-5755 USA

**Keywords:** Bevacizumab, Biosimilar, Ovarian cancer, Cervical cancer

## Abstract

**Objective:**

Bevacizumab is an important component in the treatment of various cancers, and despite guidelines recommending its use in both ovarian and cervical cancer, patient access to bevacizumab and other angiogenesis inhibitors is limited. Biosimilars are large, structurally complex molecules that are intended to be highly similar to, and treat the same condition(s) as, an existing licensed or approved (reference) biologic, with no clinically meaningful differences in purity, potency and safety. This article summarizes the role of bevacizumab in the treatment paradigm of ovarian and cervical cancer. We also discuss the potential role of biosimilars to bevacizumab, which may offer more affordable options in the future treatment of gynecologic cancers.

**Methods:**

Literature searches of PubMed and ClinicalTrials.gov databases were conducted. Regulatory and individual pharmaceutical company web pages were also reviewed. Search terms included “biosimilar” and “bevacizumab,” and these were used to identify information regarding biosimilar development, reporting results of biosimilar studies or biosimilars in development.

**Results:**

At present, four bevacizumab biosimilar candidates are undergoing comparative clinical assessment, with the potential to increase access and offer efficiencies across healthcare systems.

**Conclusions:**

It is anticipated that biologics such as bevacizumab will continue to play a key role in the treatment of an array of gynecologic cancers. Biosimilars to bevacizumab are currently in development and have the potential to increase access to medicines in a variety of settings, including gynecologic cancers.

## Introduction

Bevacizumab (Avastin®) is a recombinant humanized monoclonal immunoglobulin G1 antibody that binds to the human vascular endothelial growth factor and blocks its activity and angiogenesis [1]. Bevacizumab is the only complex biologic therapy indicated for the treatment of patients with cervical, epithelial ovarian and fallopian tube cancer in the United States (Table 1) and Europe. Bevacizumab is also approved for the treatment of patients with metastatic colorectal cancer, non-small-cell lung cancer (NSCLC) and metastatic renal cell cancer [1, 2]. Additionally, it is indicated for the treatment of patients with glioblastoma in the United States [1] and for use in metastatic breast cancer in Europe [2].

**Table 1:** Bevacizumab: approved indications in the United States [1]

Clinical indication	Combination regimen	Treatment setting
Metastatic colorectal cancer	Intravenous 5-fluorouracil–based chemotherapy	First- or second-line treatment
Metastatic colorectal cancer	Fluoropyrimidine-irinotecan– or fluoropyrimidine-oxaliplatin–based chemotherapy	Second-line treatment in patients who have progressed on a first-line bevacizumab-containing regimen
Non-squamous non-small-cell lung cancer	Carboplatin and paclitaxel	First-line treatment of unresectable, locally advanced, recurrent or metastatic disease
Glioblastoma	Monotherapy	Adult patients with progressive disease following prior therapy^a^
Metastatic renal cell carcinoma	Interferon alfa	Adult patients
Cervical cancer	Paclitaxel and cisplatin or paclitaxel and topotecan	Persistent, recurrent or metastatic disease
Platinum-resistant recurrent epithelial ovarian, fallopian tube or primary peritoneal cancer	Paclitaxel, pegylated liposomal doxorubicin or topotecan	Adult patients
Platinum-sensitive recurrent epithelial ovarian, fallopian tube or primary peritoneal cancer^b^	Carboplatin and paclitaxel or carboplatin and gemcitabine chemotherapy (followed by bevacizumab)	Adult patients who have relapsed ≥6 months following last treatment with platinum-based chemotherapy

^a^Effectiveness based on improvement in objective response rate. No data available demonstrating improvement in disease-related symptoms or survival with bevacizumab

^b^FDA approval granted on 6 Dec 2016 [3]

It is expected that the use of bevacizumab in gynecologic cancers will increase, given the recent approval (in combination with carboplatin and gemcitabine) in platinum-sensitive ovarian cancer in the United States [3] and Canada [4]. However, patient access to bevacizumab and other angiogenesis inhibitors is limited [5]. This is due to several factors, including insurance coverage, drug availability, supply and manufacturing, and concerns regarding the cost-effectiveness of bevacizumab for some patients [5].

Biologics are large, structurally complex medicinal products. Their active ingredients are created by biological processes rather than chemical synthesis. Although biologics cannot be replicated, it is possible to create a version (termed “biosimilar”) that is highly similar to an already licensed or approved reference biologic in terms of purity, safety and efficacy [6, 7]. Biosimilars have the potential to increase patient access to biologic medicines, such as bevacizumab, and this may subsequently improve clinical outcomes.

This article reviews the role of bevacizumab in the treatment paradigm of ovarian and cervical cancer. We also discuss the potential role of biosimilars to bevacizumab, which may offer more affordable options in the future treatment of gynecologic cancers.

## Review

Literature searches of PubMed and ClinicalTrials.gov databases were conducted. Regulatory and individual pharmaceutical company web pages were also reviewed. Search terms included “biosimilar” and “bevacizumab,” and these were used to identify information pertaining to biosimilar development, reporting results of biosimilar studies, or biosimilars in development.

### Bevacizumab in the treatment of gynecologic cancers: an overview

Bevacizumab, in combination with chemotherapy, is an important component of treatment of ovarian cancer. Approval of combination bevacizumab for the treatment of platinum-resistant recurrent epithelial ovarian or fallopian tube cancer was based on the results of an international, open-label, randomized study, AURELIA (Avastin Use in Platinum-Resistant Epithelial Ovarian Cancer), in patients with measurable ovarian cancer that had progressed <6 months following platinum-based treatment [8]. Median progression-free survival (PFS) was 6.7 months with bevacizumab (10 mg/kg every 2 weeks or 15 mg/kg every 3 weeks) plus chemotherapy (weekly paclitaxel, pegylated liposomal doxorubicin or topotecan) vs 3.4 months with chemotherapy alone (*P* < 0.001). Objective response rate was 27.3% with bevacizumab plus chemotherapy vs 11.8% with chemotherapy alone (*P* = 0.001). No statistically significant difference in overall survival (OS) was observed between the two treatment regimens. Hypertension and proteinuria were common adverse events in patients treated with bevacizumab plus chemotherapy [8].

The United States Food and Drug Administration (FDA) recently granted approval of bevacizumab for the treatment of platinum-sensitive recurrent epithelial ovarian, fallopian tube or primary peritoneal cancer [3]. Approval was based on the results of two randomized Phase 3 studies. The Gynecologic Oncology Group (GOG) -0213 study showed that median OS was 42.6 months with bevacizumab plus chemotherapy vs 37.3 months with chemotherapy alone (*P* = 0.056). The GOG-0213 study also showed improvement in PFS with bevacizumab plus chemotherapy (13.8 months) compared with chemotherapy alone (10.4 months; *P* < 0.0001). In the OCEANS (Ovarian Cancer Study Comparing Efficacy and Safety of Chemotherapy and Anti-Angiogenic Therapy in Platinum-Sensitive Recurrent Disease) study, median PFS was 12.4 months with bevacizumab plus chemotherapy vs 8.4 months with chemotherapy plus placebo (*P* < 0.0001). However, no statistically significant difference in OS was observed between the two treatment groups. The adverse events associated with bevacizumab in the GOG-0213 and OCEANS studies were consistent with those observed in previous studies, and included fatigue, low white blood cell count with fever, low sodium, pain in extremity, low platelet count, elevated protein in the urine, high blood pressure and headache [3]. Bevacizumab, in combination with a chemotherapy backbone, is also a key component in the treatment of cervical cancer. Approval of combination bevacizumab for the treatment of persistent, recurrent or metastatic cervical cancer was granted on the results of an international Phase 2 randomized trial [9]. Results from this study showed that bevacizumab plus chemotherapy (cisplatin plus paclitaxel or topotecan) was associated with increased OS (17.0 months) compared with chemotherapy alone (13.3 months) (*P* = 0.004). Significantly higher response rates were observed with bevacizumab plus chemotherapy (48%) compared with chemotherapy alone (36%) (*P* = 0.008). In addition, bevacizumab plus chemotherapy was associated with a higher frequency of hypertension, thromboembolic events and gastrointestinal fistulas, compared with chemotherapy alone [9]. In summary, bevacizumab in combination with chemotherapy is the mainstay of treatment for a variety of gynecologic cancers.

### Challenges and barriers to the use of bevacizumab in clinical practice

A recent retrospective population-based study using the Surveillance, Epidemiology, and End Results (SEER)-Medicare database of 9491 women with epithelial ovarian cancer showed that, despite strong evidence of improved survival associated with therapy recommended by National Comprehensive Cancer Network (NCCN) guidelines, [10] over 70% of women receiving initial treatment for epithelial ovarian cancer did not receive treatment consistent with NCCN recommendations [11]. Ultimately, this may adversely affect patient care and is a serious global concern.

Despite the clinical success of bevacizumab in cancers with a large global incidence, such as lung and colorectal cancers, and clear guidelines recommending its use in both ovarian [10] and cervical cancer, [12] there is a notable lack of patient access to bevacizumab. Disparities in access to bevacizumab and other targeted therapies have been reported in Europe, with some countries reporting only occasional access to bevacizumab, or access for only 50% of patients with ovarian cancer [5]. Therefore, it is important to identify areas of inefficiencies and to understand barriers to patient access.

Several factors, including healthcare system infrastructure, stage at diagnosis, population health and lifestyle and availability of anticancer agents, can influence access to cancer therapy. Issues related to insurance coverage, treatment cost, drug availability, supply and manufacturing may create barriers to the use of bevacizumab in many countries worldwide. A survey conducted by the European Society of Medical Oncology (ESMO) Consortium reported budget and affordability issues, and problems with the manufacture and supply of bevacizumab as the most common factors leading to suboptimal access to bevacizumab in a variety of cancers [5].

Clinical trials demonstrated bevacizumab improves PFS in patients with ovarian cancer [8] and OS in cervical cancer [9]. Although bevacizumab with chemotherapy is more effective with regard to PFS than chemotherapy alone, it is not a cost-effective, front-line treatment regimen in the overall population of patients with ovarian cancer (Table 2) [13]. Furthermore, approximately three-quarters of US oncologists do not consider bevacizumab a “good value” treatment option [14]. However, a recent analysis utilized results from the AURELIA study of bevacizumab plus chemotherapy versus chemotherapy alone in patients with platinum-resistant recurrent ovarian cancer [8]. This analysis concluded that bevacizumab was cost-effective in this setting [15]. Taken together, it is clear that further studies are needed to determine the cost-effectiveness of bevacizumab in the real-world setting.

**Table 2:** Cost-effectiveness of bevacizumab in the front-line treatment of ovarian cancer [13]

Citation	Treatment regimen	Total/Incremental costs (USD)	Effectiveness/Incremental effectiveness	ICER	Key findings
Cohn et al. 2011	PAC + CAR	2.5 million^a^	10.3 months^b^	Referent	Addition of BEV and maintenance BEV was not cost-effective
	PAC + CAR + BEV	21.4 million^a^	11.2 months^b^	USD479,712 per PFLY gained	
	(PAC + CAR + BEV) + maintenance BEV	78.3 million^a^	14.1 months^b^	USD401,088 per PFLY gained	
Barnett et al. 2013	PAC + CAR PAC + CAR + BEV PAC + CAR + BEV for high-risk patients	6220^c^ 20,751^c^ 56,351^c^	2.80^d^ 2.89^d^ 2.88^d^	Referent USD168,610 per QALY Dominated	Use of BEV with standard first-line taxane was not cost-effective in stage III/IV ovarian cancer. May be suitable in high-risk patients although ICER exceeded thresholds
Chan et al. 2014	PAC + CAR	535^e^	10.5^b^	Referent	For high-risk, advanced ovarian cancer patients, ICER was almost USD170,000 per life-year saved
	PAC + CAR + BEV plus maintenance BEV	3760 (3225 for maintenance)^e^	15.9^b^	USD167,771 per LYG	

*BEV* bevacizumab; *CAR* carboplatin; *ICER* incremental cost-effectiveness ratio; *LYG* life-year gained; *PAC* paclitaxel; *PFLY* progression-free life-year; *QALY* quality-adjusted life-year; *USD* United States dollars

^a^Total cost for 600 patients

^b^Median progression-free survival

^c^Mean cost

^d^QALY

^e^Total cost per cycle

Dominated: BEV was more costly and less effective

Bevacizumab will continue to remain an important component in the treatment of gynecologic cancers as well as other settings. In light of the limited access to bevacizumab worldwide, additional treatment options for gynecologic cancers are eagerly awaited.

### Development of biosimilars and their potential benefits

Patents for bevacizumab will shortly expire in the United States and Europe [16]. Biosimilars are large, structurally complex molecules that are intended to be highly similar to, and treat the same condition(s) as, an existing licensed or approved (reference) biologic [6, 7]. Biosimilars may offer increased treatment options for patients and physicians and have the potential to optimize efficiencies across healthcare systems worldwide. Additionally, biosimilars may provide lower cost alternatives and therefore increase access to biologics and allow greater use of biologic therapies, which may facilitate improved clinical outcomes.

**Fig. 1:**
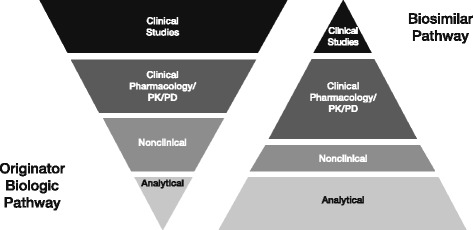
Development pathways for originator biologics and biosimilars: a different way of thinking. Adapted from Kozlowski et al., 2012. *PD*: pharmacodynamics, *PK*: pharmacokinetics

The aim of biosimilar development is not to re-establish efficacy and safety, but to demonstrate similarity to the reference biologic in terms of quality, safety and efficacy (Fig. 1) [6, 7]. The development of biosimilars involves biochemical, biophysical and functional comparative studies, and detailed characterization of the potential biosimilar. Together with comparative nonclinical, pharmacokinetic (PK), and comparative clinical trials, these data comprise the “totality of the evidence” [6].

Biosimilars must have an identical primary amino acid sequence and the same route of administration, strength and type of administration as the reference biologic [6, 7]. Biosimilars are manufactured through a process of reverse engineering and must undergo extensive comparative structural and functional characterization using state-of-the-art technology and highly specialized techniques to identify any differences between the proposed biosimilar and the reference biologic, particularly those that may alter the mechanism of action [17].

A series of analytical similarity assessments are conducted to confirm identical amino acid sequences, similar post-translational modifications and highly similar biologic activity between the proposed biosimilar and the reference biologic. Analytical similarity forms the foundation for similarity in safety and efficacy. In addition, a comprehensive assessment of the structural and functional similarity of the potential biosimilar and the reference biologic is conducted using state-of-the-art techniques, physicochemical methods and functional assays [17].

Regulatory agencies do not typically require extensive nonclinical studies for the approval of biosimilars, although this is assessed on a case-by-case basis [6, 7]. A comparative clinical study is generally conducted in one therapeutic indication to demonstrate that there are no clinically meaningful differences in PK, pharmacodynamics (PD), efficacy or safety, including immunogenicity, between the potential biosimilar and the reference biologic. The goal of a comparative clinical study is to address any residual uncertainty between the proposed biosimilar and the reference biologic [6, 7]. Because all biologics, including biosimilars, have the potential to trigger an immunogenic response, which may alter the PK, efficacy or safety properties, [18] the formation of antidrug antibodies is carefully monitored throughout development and during postmarketing surveillance.

### Biosimilars and the scientific basis of extrapolation across indications

Extrapolation is a scientific and regulatory principle that describes the approval of a biosimilar for use in an indication held by the reference biologic, which is not directly studied in a comparative clinical trial with a biosimilar. Extrapolation is key to the concept of biosimilarity and is based on establishing a similar mechanism of action for the biosimilar in various disease indications [6]. As well as reducing or eliminating the need for studies in multiple indications, extrapolation can potentially allow greater access to biosimilars, with minimal delays in treatment. The concept of extrapolation is supported by the US FDA and the European Medicines Agency (EMA) regulatory guidelines [6, 7]. However, the decision to extrapolate data from one indication to another is made on a case-by-case basis, with strong scientific justification and the totality of evidence.

The mechanism of action of bevacizumab involves the inhibition of vascular endothelial growth factor (VEGF), which has an important role in tumor angiogenesis and vascularization [1, 2]. Bevacizumab is an effective treatment for a number of tumors and its mechanism of action is independent of tumor site [1]. This forms the basis of the scientific rationale for extrapolation of similarity data across indications and may support the approval of bevacizumab biosimilars in indications held by the reference biologic without clinical studies in gynecologic indications.

### Bevacizumab biosimilar candidates in development

Four bevacizumab biosimilar candidates have completed preclinical assessments and, based on the totality of evidence, are currently undergoing comparative clinical assessments (Table 3). ABP 215 (Amgen) showed similar in vitro functional characteristics and equivalent human PK to bevacizumab [19] and demonstrated clinical equivalence and similar safety and immunogenic profiles as bevacizumab in patients with non-squamous NSCLC [20]. BCD-021 (Biocad) showed similar PK and safety to bevacizumab in patients with NSCLC [21]. BCD-021 also demonstrated similar efficacy, safety and immunogenicity to bevacizumab in patients with advanced non-squamous NSCLC [22]. A multicenter, randomized, double-blind clinical trial is ongoing to evaluate the efficacy and safety of BI 695502 (Boehringer Ingelheim) compared with bevacizumab (in combination with chemotherapy) in patients with advanced non-squamous NSCLC (ClinicalTrials.gov, NCT02272413). PF-06439535 (Pfizer) showed a similar structure and in vitro biological activity, and a similar in vivo toxicologic and toxicokinetic profile as bevacizumab [23, 24]. PF-06439535 also demonstrated PK similarity and comparable safety profiles to bevacizumab in healthy male volunteers [25]. A trial of PF-06439535 vs bevacizumab sourced in the EU in patients with advanced non-squamous NSCLC who have not received prior chemotherapy is ongoing (ClinicalTrials.gov, NCT02364999).

**Table 3:** Bevacizumab biosimilars in late-stage clinical development

Sponsor	Biosimilar candidate	Patient population	Study objectives	Key findings
Amgen	ABP 215	NSCLC	Functional similarity and PK equivalence	•Similar functional characteristics •Equivalent PK [26]
		NSCLC	Clinical equivalence of objective response rate	•Clinical equivalence •Similar safety and immunogenic profiles to bevacizumab [20]
Biocad	BCD-021	NSCLC	PK and safety	•Similar PK and safety [21]
		NSCLC	Overall response rate	•Similar efficacy, safety and immunogenicity [22]
Boehringer Ingelheim	BI 695502	NSCLC	Efficacy and safety	•Recruiting (NCT02272413)
		mCRC	Efficacy and safety	•Recruiting (NCT02776683)
Pfizer	PF-06439535	NSCLC	Nonclinical evaluation	•Similar structure and in vitro biological activity [24] •Similar in vivo toxicology [24]
		NSCLC	PK and safety	•PK similarity [25] •Comparable safety profile [25]
		NSCLC	Comparative efficacy and safety	•Ongoing (NCT02364999)

*NSCLC* non-small-cell lung cancer; *PK* pharmacokinetics

## Conclusions

It is anticipated that biologics such as bevacizumab will continue to play a key role in the treatment of an array of gynecologic cancers. Limited access to bevacizumab and the lack of cost-effectiveness in some patients has driven the need to develop safe and effective biosimilars to bevacizumab, which have the potential to increase access to medicines and offer efficiencies across healthcare systems.

## Abbreviations

AURELIA: Avastin Use in Platinum-Resistant Epithelial Ovarian Cancer
EMA: European Medicines Agency
ESMO: European Society of Medical Oncology
FDA: Food and Drug Administration
GOG: Gynecologic Oncology Group
NCCN: National Comprehensive Cancer Network
NSCLC: non-small-cell lung cancer
OCEANS: Ovarian Cancer Study Comparing Efficacy and Safety of Chemotherapy and Anti-Angiogenic Therapy in Platinum-Sensitive Recurrent Disease
OS: Overall survival
PFS: Progression-free survival
SEER: Surveillance, Epidemiology, and End Results
VEGF: Vascular endothelial growth factor.
